# cN1A Antibody-Positive Inclusion Body Myositis Following Seminoma Manifesting With Slowly Progressive Paraparesis: A Case Report

**DOI:** 10.7759/cureus.101163

**Published:** 2026-01-09

**Authors:** Josef Finsterer

**Affiliations:** 1 Neurology, Neurology and Neurophysiology Center, Vienna, AUT

**Keywords:** cn1a antibodies, myositis, myositis-associated antibodies, paraneoplastic syndrome, paraparesis

## Abstract

A case of inclusion body myositis (IBM) with autoantibodies against cN1A following orchiectomy for seminoma has not been previously described. A 60-year-old man developed slowly progressive, painless paraparesis of the lower extremities and mild hyper-creatine kinase (CK)emia three years after orchiectomy for a unilateral seminoma. Clinical examination 10 years after onset revealed weakness of hip flexion (Medical Research Council (MRC) 5-), knee flexion (MRC 5-), and foot extension (left MRC 4-, right MRC 5-); absent tendon reflexes; and thigh muscle atrophy. Needle electromyography was normal, but contrast-enhanced muscle MRI indicated myositis. Quadriceps muscle biopsy revealed terminally remodeled muscle tissue with fibrosis and adipose tissue replacement, as well as a few residual, markedly atrophic, myopathic muscle fibers and COX-negative fibers suggestive of IBM, primary myopathy, or a neurogenic lesion with secondary myopathy. Myositis antibody testing showed a marked increase in cN1A. In summary, this case demonstrates that cN1A antibody-associated IBM can occur following seminoma, IBM can begin with proximally accentuated paraparesis of the lower extremities, the correct diagnosis can take years, and muscle MRI, biopsy, and antibody testing, but not electromyography, can be diagnostically helpful.

## Introduction

Inclusion body myositis (IBM) is an acquired, age-related, idiopathic myopathy characterized by degenerative pathogenesis [1]. IBM typically develops after the age of 50 years and affects men more often than women. While inflammatory cells are often present in muscle biopsies of patients with IBM, these inflammatory infiltrates are considered secondary changes. Although IBM can be associated with malignant diseases such as chronic lymphatic leukemia and T-cell large granular lymphocytic leukemia, there is no broad association with an increased incidence of malignancies, which is why it is not regarded as paraneoplastic [1]. The muscles most commonly affected in IBM are the wrist and finger muscles (flexor carpi radialis, flexor carpi ulnaris, palmaris longus (wrist flexion), flexor digitorum superficialis, flexor digitorum profundus, flexor pollicis longus (finger flexion)), as well as the muscles of the quadriceps. Extension of the foot may also be affected [2]. Less common symptoms include isolated dysphagia, asymptomatic hyper-creatine kinase (CK)emia, and axial or limb weakness beyond the typical pattern [2]. IBM is associated with significant morbidity, as most patients eventually require a wheelchair and exhibit impaired hand function and severe dysphagia [2]. The disease progresses at different rates and with different symptoms in each person, but generally has little impact on life expectancy and lifespan, with aspiration pneumonia and respiratory complications being the most common causes of death [2]. IBM is diagnosed based on the clinical presentation (weakness of the finger flexors, quadriceps), increased CK levels, electromyography, muscle MRI, muscle biopsy (inflammation, rimmed vacuoles, proteon aggregates, mitochondrial changes), specific autoantibodies, and the exclusion of differential diagnoses.

cN1A antibodies are used as a biomarker for IBM and are directed against the cytoplasmic 5'-nucleotidase 1A protein (cN1A). They can help differentiate IBM from other forms of myositis [3]. Although they are not detectable in all patients (sensitivity, 30-70%), they have adequate specificity; however, false-positive results may occur depending on the testing laboratory [3]. cN1A can also be found in polymyositis and dermatomyositis, Sjögren’s syndrome, systemic lupus erythematosus, amyotrophic lateral sclerosis, and post-polio syndrome [4]. However, they occur less frequently in these other diseases than in IBM, and their specificity for IBM may be reduced in the presence of these other diseases [4]. cN1A antibody-positive IBM has been associated with autoimmune disease, but a case of cN1A antibody-positive IBM following orchidectomy for seminoma has not yet been described.

## Case presentation

**Figure 1:**
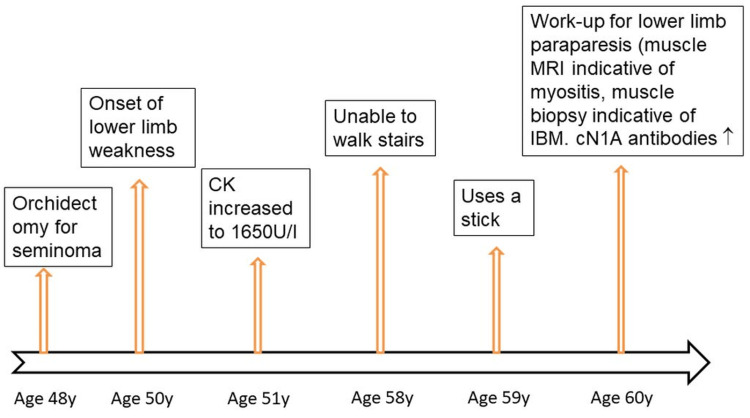
Chronological progression of the disease. CK = creatine kinase; IBM = inclusion body myositis

**Figure 2:**
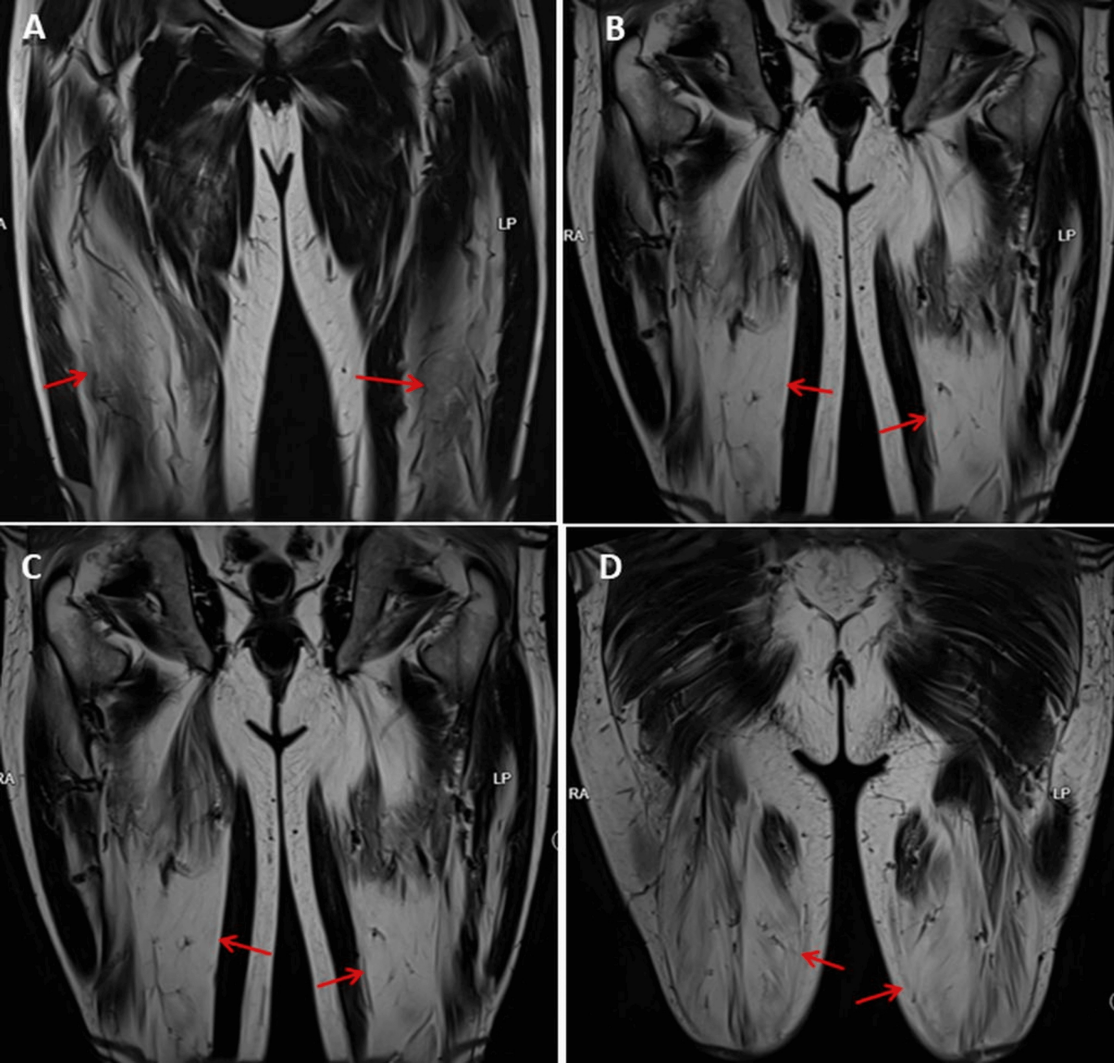
Muscle MRI (T2-weighted imaging, coronary view, Dixon technique, fat suppression) showing hyperintensity (fatty infiltration) in the vastus intermedius (A, arrows), rectus femoris (B, arrows), vastus lateralis (C, arrows), and hamstring muscles (D, arrows).

**Figure 3:**
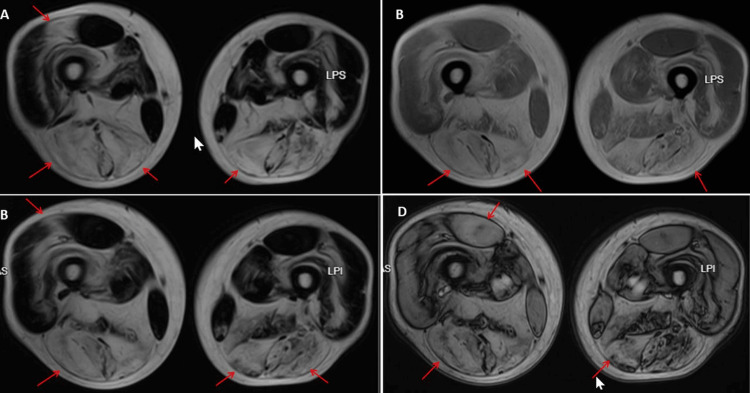
Muscle MRI (T1-weighted imaging, axial view Dixon technique pre in F (A), pre in R (B)) showing hyperintensity (fatty infiltration) in the hamstrings and rectus femoris muscles (arrows), which did not enhance on contrast medium (C, D, arrows).

A 60-year-old man developed slowly progressive, bilateral, painless, proximally accentuated weakness of the lower extremities at the age of 50 years. His medical history included a seminoma, which had been treated by orchiectomy at the age of 48 years, right-sided glaucoma, astigmatism, arterial hypertension, and hyperlipidemia (Figure 1). In the first two years after the orchiectomy, he was examined for relapse every three months. Three years after the orchiectomy, at the age of 51 years, CK was tested due to lower limb muscle weakness that had been present for a year. Slightly elevated values, up to 1,650 U/L, were repeatedly found. He was unable to climb stairs from the age of 58 years (Figure 1). Evaluation of the paraparesis and the recurrent hyper-CKemia revealed proximally accentuated weakness of hip flexion (Medical Research Council (MRC) 5-), knee flexion (MRC 5-), and foot extension (MRC 4- left, MRC 5- right); thigh muscle atrophy; and diminished tendon reflexes of the lower extremities. Needle electromyography and nerve conduction velocity studies were normal. Muscle MRI of the lower extremities showed evidence of the stage of fatty infiltration of polymyositis (Figures 2, 3). Testing for myositis-specific and myositis-associated antibodies revealed markedly elevated cN1A antibodies (enzyme-linked immunosorbent assay, commercial laboratory). Repeated measurements of cN1A antibodies showed an increase each time. Muscle biopsy of the right lateral vastus muscle showed terminally remodeled muscle tissue with fibrosis and adipose tissue replacement, as well as a few residual, severely atrophic myopathic muscle fibers. NADH staining showed some target-like fibers, while COX staining showed several negative fibers. This suggested advanced IBM, a primary myopathy, or an advanced neurogenic lesion with secondary myopathy. The patient was advised to undergo glucocorticoid therapy, which he declined due to potential long-term side effects. He also declined rituximab therapy at the time. To rule out Becker muscular dystrophy and limb-girdle muscular dystrophy, he was referred to human genetics. The test results are not yet available. Only McArdle disease has been genetically ruled out so far. During the last follow-up examination, the patient reported for the first time muscle pain at the end of his workday.

## Discussion

The presented patient is interesting for several reasons. First, he is the first to develop cN1A antibody-positive IBM following seminoma, to our knowledge. Previously, cN1A antibody-positive IBM has been described in patients with Sjögren’s syndrome [5], GAD65-positive encephalitis [6], motor neuron disease [4], interstitial lung disease [7], polymyositis, dermatomyositis, systemic lupus erythematosus [8], and post-polio syndrome [4]. In connection with malignant diseases, cN1A antibodies have been reported in patients with papillary thyroid carcinoma [9], but never before in patients with seminoma.

Second, the patient did not present with the classic picture of IBM (weakness of the hand and finger extensors, hip flexors, and foot extensors), but initially with mild weakness of hip flexors and knee extensors. Later, he also developed an asymmetrical weakness in foot extension. At first, he was still able to walk without assistance, but as his weakness increased, he needed a walking stick, could no longer climb stairs, and planned to give up his work as a craftsman. He also complained of weakness in his right arm, but repeated clinical neurological examinations have so far revealed no muscle weakness in his right arm. At the last follow-up examination, he also reported muscle soreness in his legs after work.

Third, the patient presented with only weakness and muscle wasting, but without myalgia or sensory disturbances. Myalgia is a less common symptom of IBM [10]. Most IBM patients report no myalgia [10]. In general, the clinical picture of IBM involves slowly progressive muscle weakness, typically beginning after the age of 50 years and often going unnoticed due to the aging process. Both proximal (legs, hips) and distal (wrists, fingers) muscles are affected. Characteristic features include difficulty rising from a chair, stumbling, and loss of grip strength, accompanied by dysphagia and muscle wasting, usually asymmetrically, which distinguishes IBM from other inflammatory myopathies [10]. Muscle pain or cramps may occur, but are less common and usually mild [10].

Fourth, it took 10 years to diagnose cN1A-associated IBM in the index patient. Although IBM is often diagnosed late, especially with an atypical clinical presentation, 10 years, as in the index patient, is an exceptionally long time. This delay could be explained by the atypical presentation of mildly progressive lower extremity paraparesis, the absence of significant muscle pain, the failure to recognize persistent hyper-CKemia, and the lack of consideration of IBM as a differential diagnosis for paraparesis.

cN1A antibodies have been repeatedly reported in IBM patients and are found in about half of these patients. A 31-year-old female patient with Sjögren's syndrome and lymphatic leukemia presented with proximal weakness of the lower extremities that had been present for two years and an elevated serum CK level of 1,117 U/L [11]. A muscle biopsy revealed polymyositis [1]. The patient received prednisolone, methotrexate, and chloroquine, but without success [1]. After the diagnosis was changed to IBM, she was switched to rituximab, which led to significant improvement [11]. cN1A-associated IBM has also been described in several other patients; however, these studies lack information on demographics, clinical presentation, laboratory and biopsy findings, and treatment (Table 1) [5,11-13]. cN1A antibodies support the diagnosis of IBM, but are not pathognomonic [8]. They bind to 5' nucleosidase 1A (NT5c1A), which plays a key role in purine metabolism.

**Table 1:** Patients with IBM and positive cN1A antibodies reported in the literature to date. CK = creatine-kinase; IBM = inclusion body myositis; MTX = methotrexate; NR = not reported; PM = polymyositis; RTX = rituximab

Age	Sex	CK	Biopsy	Comorbidity	Treatment	Reference
60	Male	1,650	IBM	Seminoma	None	Index case
31	Female	473	PM, IBM	Sjögren’s syndrome, leukemia	Prednislone, MTX, RTX	Limaye et al. (2020) [11]
N = 3	NR	NR	Myositis	Sjögren’s syndrome	NR	Levy et al. (2022) [5]
N = 88	NR	NR	IBM	NR	NR	Herbert et al. (2016) [12]
N = 71	20 females	NR	IBM	NR	NR	Lloyd et al. (2016) [13]

## Conclusions

In summary, this case demonstrates that diagnosing IBM can be challenging years after disease onset and relies primarily on clinical presentation, detection of myotitis-specific antibodies, muscle MRI, and exclusion of various differential diagnoses. In the chronic stage, nerve conduction velocity measurements, electromyography, and biopsies are less helpful. The case also highlights that IBM can begin with proximally accentuated paraparesis of the lower extremities, correct diagnosis can take years, and the distal muscles of the upper extremities may not be primarily affected. The case expands the spectrum of clinical manifestations of IBM. Further studies are needed to investigate the significance of cN1A antibodies in the diagnosis and pathogenesis of IBM.
